# P53 regulates disruption of neuronal development in the adult hippocampus after irradiation

**DOI:** 10.1038/cddiscovery.2016.72

**Published:** 2016-10-03

**Authors:** Y-Q Li, ZW-C Cheng, SK-W Liu, I Aubert, C S Wong

**Affiliations:** 1 Department of Radiation Oncology, Sunnybrook Health Sciences Centre, University of Toronto, Toronto, ON, Canada; 2 Institute of Medical Science, University of Toronto, Toronto, ON, Canada; 3 Department of Laboratory Medicine and Pathobiology, Sunnybrook Health Sciences Centre, University of Toronto, Toronto, ON, Canada

## Abstract

Inhibition of hippocampal neurogenesis is implicated in neurocognitive dysfunction after cranial irradiation for brain tumors. How irradiation results in impaired neuronal development remains poorly understood. The *Trp53* (*p53*) gene is known to regulate cellular DNA damage response after irradiation. Whether it has a role in disruption of late neuronal development remains unknown. Here we characterized the effects of p53 on neuronal development in adult mouse hippocampus after irradiation. Different bromodeoxyuridine incorporation paradigms and a transplantation study were used for cell fate mapping. Compared with wild-type mice, we observed profound inhibition of hippocampal neurogenesis after irradiation in mice deficient in p53 despite the absence of acute apoptosis of neuroblasts. The putative neural stem cells were apoptosis resistant after irradiation regardless of *p53* genotype. Cell fate mapping using different bromodeoxyuridine incorporation paradigms revealed enhanced activation of neural stem cells and their consequential exhaustion in the absence of p53 after irradiation. Both *p53*-knockout and wild-type mice demonstrated similar extent of microglial activation in the hippocampus after irradiation. Impairment of neuronal differentiation of neural progenitors transplanted in irradiated hippocampus was not altered by *p53* genotype of the recipient mice. We conclude that by inhibiting neural progenitor activation, p53 serves to mitigate disruption of neuronal development after irradiation independent of apoptosis and perturbation of the neural stem cell niche. These findings suggest for the first time that p53 may have a key role in late effects in brain after irradiation.

Radiotherapy is an important cancer treatment modality for primary and secondary brain tumors. Unfortunately, cranial irradiation may result in devastating late clinical consequences including neurocognitive impairment.^1^ Although recent advances in radiation planning and delivery have allowed for a reduction in the volume of normal brain irradiated, whole or large volume brain irradiation remains the standard treatment for multiple brain metastases and many intracranial tumors.

Multipotent neural progenitor cells (NPCs) or stem cells are present in adult mammalian brain. They continuously generate new neurons, a process termed neurogenesis. An area in adult mammalian brain where neurogenesis has been characterized is the dentate gyrus of the hippocampus. Radial glial cells, or type-1 cells, in the subgranular zone (SGZ) of the dentate gyrus are thought to be the neural stem cells. Once activated, they undergo asymmetric divisions to self-renew and generate proliferative type-2 NPCs or intermediate neural progenitors (INPs). INPs give rise to type-3 NPCs or neuroblasts, which differentiate into immature and then mature neurons that become integrated into the neuronal circuitry.^2,3^

Neurogenesis is associated with hippocampal function of learning and memory.^4–7^ Irradiation is known to disrupt neurogenesis,^8^ a process implicated in neurocognitive decline following cranial irradiation.^9^ Damage of the vascular niche for neurogenesis is thought to contribute to inhibition of neuronal development after irradiation.^1^

The *Trp53* (*p53*) gene has a major role in regulating cellular response after irradiation.^10^ Alterations in the *p53* gene have been linked to tumor resistance to radiotherapy. There is evidence that p53 has a role in regulating radiation injury in the gastrointestinal tract and the heart.^11,12^ Enhanced anticancer effects have also been shown by genetic and pharmacologic inhibition of p53 in tumor endothelium.^13^ Whether and how p53 regulates inhibition of adult neurogenesis after irradiation is unclear. Here we showed that deficiency in p53 resulted in enhanced activation of neural stem cells and NPCs, with consequential depletion of the neural stem cell pool and profound inhibition of neurogenesis after irradiation. These findings provide novel mechanistic insight into the molecular regulation of disruption of hippocampal neuronal development after irradiation.

## Results

### DNA damage response is altered in p53-deficient NPCs

We first asked whether DNA damage response following irradiation in NPCs was altered in the absence of p53. The kinetics of formation and loss of *γ*H2AX nuclear foci is associated with the efficiency of repair of DNA strand breaks and radiosensitivity.^14^ We thus used *γ*H2AX nuclear foci as a readout for DNA damage response in NPCs cultured from mice, wild type (+/+) or knock out (−/−), of the *p53* gene. Consistent with the negative effects of p53 on cell proliferation,^15,16^ neurospheres generated from *p53*−/− mouse brain grew faster and were larger compared with those derived from *p53*+/+ mice. Dissociated neurosphere *p53*−/− cells cultured in non-differentiation medium also demonstrated a higher density compared with *p53*+/+ cells. These cells were positive (+) for nestin and sex-determining region Y-box 2 (SOX2), markers of early NPCs (Supplementary Figures 1a–f).

NPCs cultured from dissociated neurosphere showed only the occasional *γ*H2AX nuclear foci. At 1 h after 5 Gy, there was a marked increase in nuclear foci (Supplementary Figures 1g–j). The number of foci per nucleus returned to non-irradiated level by 24 h. Compared with *p53*+/+ NPCs, there was delay in clearance of *γ*H2AX nuclear foci in *p53*−/− NPCs at 3 h after irradiation, and the effect of p53 was independent of time after irradiation (number of foci per nucleus: time after irradiation, *P*<0.0001; *p53* genotype, *P*<0.0001; interaction, *P*=0.0001; % nuclei with foci; time after irradiation, *P*<0.0001; *p53 *genotype, *P*<0.01; interaction, *P*=0.0001; two-way analysis of variance (ANOVA); Supplementary Figures 1k–l). These results were consistent with altered DNA damage response in NPCs *in vitro* after irradiation in the absence of p53.

### Deficiency in p53 results in profound inhibition of neurogenesis after irradiation

**Figure 1:**
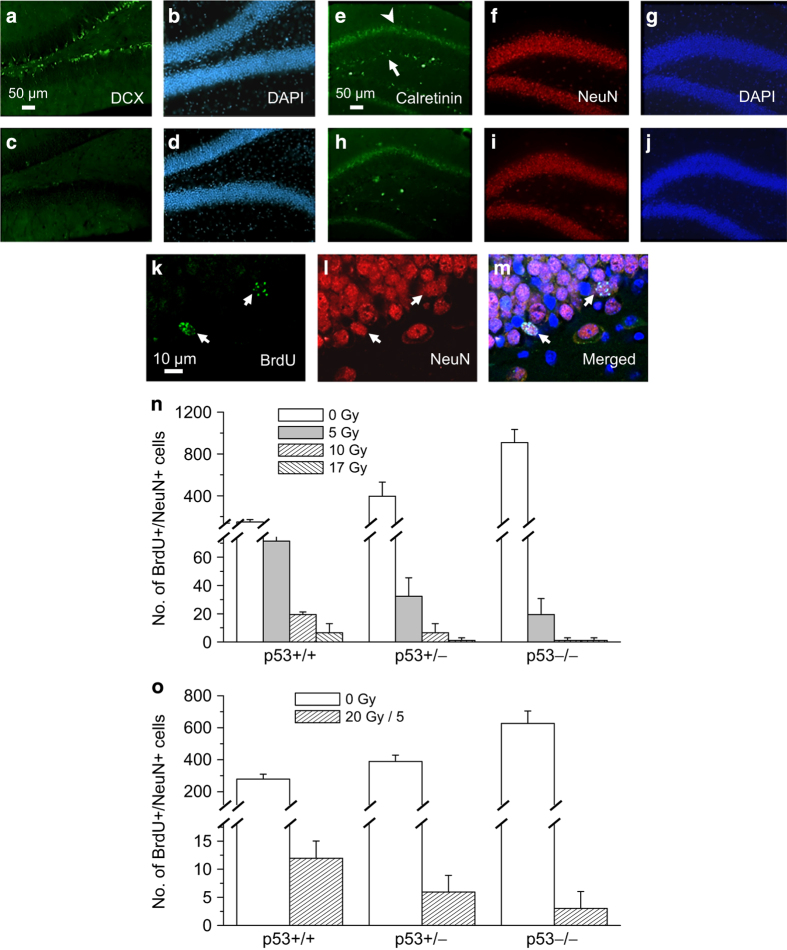
Inhibition of hippocampal neurogenesis after irradiation is p53 dependent. There is loss of DCX+ (**a** and **b**, 0 Gy; **c** and **d**, 17 Gy; DCX, green; DAPI, blue) and calretinin+ cells (**e**–**g**, 0 Gy, **h–j**, 17 Gy; calretinin cells, arrow, green; NeuN, red; DAPI, blue) in SGZ at 9 weeks after irradiation. Arrowhead (**e**) denotes the normal band of calretinin+ nerve fibers at the inner molecular layer. Newborn neurons in dentate gyrus demonstrate BrdU (**k**, arrows, green) and NeuN immunostaining (**l**, red; **m**, merged). The *p53* genotype has an independent effect on the number of BrdU+/NeuN+ cells at 9 weeks after single doses of cranial irradiation (**n**) or 20 Gy in 5 daily fractions (**o**). Mice were given BrdU daily for 7 consecutive days 4 weeks after irradiation. Data are expressed as mean±S.E.M. and analyzed with two-way ANOVA with three to five mice per dose per genotype.

Irradiation is known to inhibit hippocampal neurogenesis.^8^ At 9 weeks after irradiation, a very apparent change in dentate gyrus was the marked loss of cells immunoreactive for doublecortin (DCX) and calretinin, markers of neuroblasts and immature neurons, respectively (DCX+ cells, 315±104, 17 Gy *versus* 9896±483, 0 Gy, *P*<0.00001, *t*-test (Figures 1a–d); calretinin+ cells, 423±12, 17 Gy *versus* 910±188, 0 Gy, *P*<0.05 (Figures 1e–j)).

To determine directly the effects of irradiation on neurogenesis, mice were given bromodeoxyuridine (BrdU), 50 mg/kg daily x7 days, 4 weeks after irradiation. Animals were killed 9 weeks after irradiation for an analysis of the number of newborn neurons or BrdU+ cells immunoreactive for the neuronal marker, neuronal nuclei (NeuN) (Figures 1k–m). Irradiation resulted in a dose-dependent decrease in the number of BrdU+/NeuN+ cells. Consistent with the negative effect of p53 in cell proliferation,^15,16^ an increase in BrdU+/NeuN+ cells was associated with p53 deficiency. In contrast, the number of BrdU+/NeuN+ cells after irradiation demonstrated the opposite effect, highest in *p53*+/+ mice, intermediate in *p53* heterozygous (+/−) mice and lowest in *p53*−/− mice (radiation dose, *P*<0.0001; *p53* genotype, *P*<0.0005; interaction, *P*<0.0001, two-way ANOVA; Figure 1n). Results of pairwise comparisons are shown in Supplementary Table 1. The number of BrdU+/NeuN+ cells after 5 Gy in *p53*+/+, *p53*+/− and *p53*−/− mice decreased to 50.6%, 10.4% and 1.9%, respectively, compared with their respective genotype controls (Supplementary Figure 2). This profound inhibition of neurogenesis associated with p53 deficiency was also observed after a clinically relevant irradiation schedule of 20 Gy in 5 daily fractions (irradiation, *P*<0.0001; *p53* genotype, *P*<0.005; interaction, *P*<0.005; Figure 1o).

To determine whether an extra copy of *p53* gene conferred protection, neurogenesis in super-*p53* (*p53*^*S*^) mice that have an extra copy of *p53* gene^17^ was compared with their wild-type littermates after irradiation. The number of BrdU+/NeuN+ cells was significantly reduced in both *p53*^*S*^ mice and wild-type controls after 5 Gy, but there was no evidence of a protective effect because of the extra copy of *p53* gene (irradiation, *P*<0.005; *p53*^*S*^ genotype, *P*-value not significant; Supplementary Figure 3).

### P53 regulates impairment of neurogenesis after irradiation independent of apoptosis of neuroblasts

NPCs in the SGZ of dentate gyrus are known to undergo apoptosis within hours of irradiation.^18,19^ It has been postulated that apoptosis of NPCs contributes to impaired neurogenesis after irradiation.^18^ In non-irradiated *p53*+/+ mice, apoptotic cells were rarely observed in the SGZ, a robust apoptotic response in the SGZ within hours after irradiation as shown previously.^19,20^ The peak response, 9849±622, of apoptotic cells based on the morphologic criteria was observed at 8 h after irradiation, compared with 91±27 in control (*P*<0.001, *t*-test). The response returned to non-irradiated control level by 24 h. Similar results were observed using terminal deoxynucleotidyl transferase dUTP nick-end labeling (TUNEL) and caspase-3 immunohistochemistry (data not shown).

**Figure 2:**
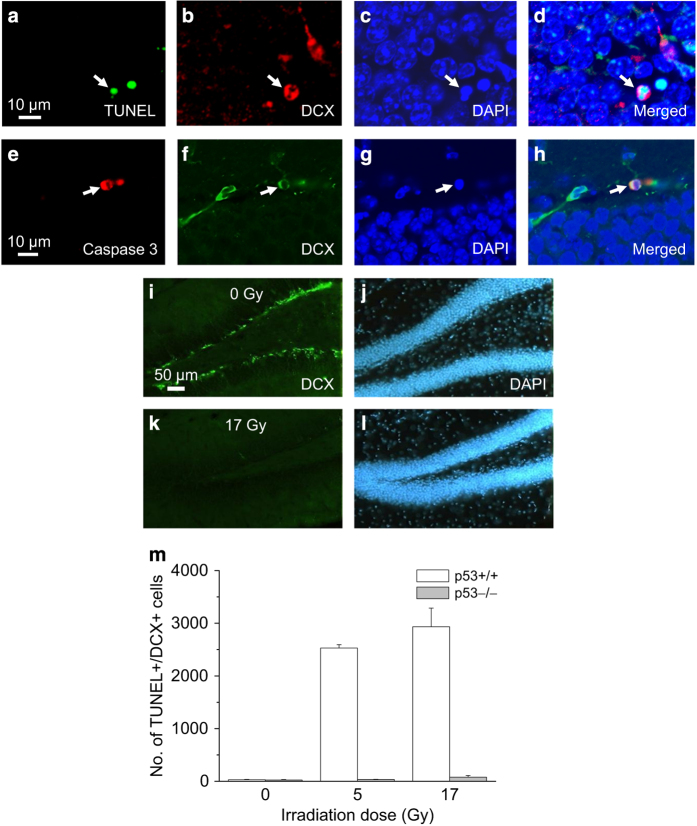
Neuroblasts in SGZ undergo p53-dependent apoptosis after irradiation. DCX+ apoptotic cells are identified using TUNEL (**a–d**, arrows) and caspase-3 immunohistochemistry (**e–h**, arrows). There is a marked loss of DCX+ cells at 24 h after irradiation (**i** and **j**, 0 Gy; **k** and **l**, 17 Gy; DCX, green; DAPI, blue). The number of DCX+/TUNEL+ apoptotic cells observed at 8 h is radiation dose and *p53* genotype dependent. Data are expressed as mean±S.E.M. and analyzed with a two-way ANOVA with three to five mice per experimental group.

Of the apoptotic cells that showed characteristic nuclear condensation and fragmentation, about a third expressed DCX. Among the TUNEL+ and caspase-3+ cells, about a third also expressed DCX (Figures 2a–h). None of the DCX+ apoptotic cells expressed nestin. Consistent with DCX-expressing cells or neuroblasts representing the apoptosis-susceptible population after irradiation, a marked clearance of DCX+ cells was observed at 24 h after irradiation (5553±2126, 17 Gy *versus* 21 773±1598, 0 Gy, *P*<0.005, *t*-test; Figures 2i–l).

Type-1 cells express glial fibrillary acidic protein (GFAP) and nestin, and have a characteristic long radial process that spans the entire granule cell layer and ramifies in the molecular layer.^2,3^ Although the occasional apoptotic cells expressed nestin, no GFAF+/nestin+ apoptotic cells were observed. At 24 h after irradiation, the number of GFAP+/nestin+ cells remained unchanged (1851±179, 17 Gy *versus* 1743±150, 0 Gy, *t*-test, *P*-value not significant). These results provide no evidence that type-1 cells undergo radiation-induced apoptosis.

Radiation-induced apoptosis of subgranular cells is known to be p53 dependent.^21,22^ It was extremely difficult to observe apoptotic cells in *p53*−/− mice after irradiation. Following irradiation, the number of TUNEL+/DCX+ cells at 8 h was dose and *p53* genotype dependent (irradiation dose, *P*<0.001; *p53* genotype, *P*<0.001, two-way ANOVA; Figure 2m). Abrogation of radiation-induced apoptosis in *p53*−/− mice supports the notion that p53 regulates inhibition of neurogenesis after irradiation independent of acute apoptosis of neuroblasts.

### Irradiation results in p53-dependent late ablation of proliferating, newborn and total neural stem cells

**Figure 3:**
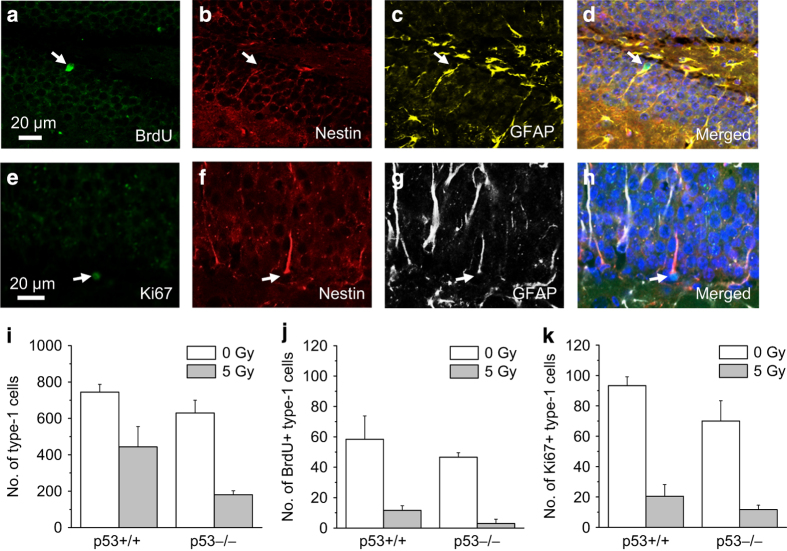
Irradiation results in p53-dependent ablation of type-1 cells in mouse dentate gyrus. A representative newborn type-1 cell (**a–d**, arrow) demonstrates BrdU incorporation (**a**, green), and is positive for nestin (**b**, red) and GFAP (**c**, yellow; **d**, merged), and has a characteristic process that traverses the granule cell layer. A proliferating type-1 cell (**e–h**, arrow) demonstrates immunostaining for Ki67 (**e**, green), nestin (**f**, red) and GFAP (**g**, white; **h**, merged). At 9 weeks after irradiation, there is p53-dependent reduction of total (**i**), BrdU+ (**j**) and Ki67+ type-1 cells (**k**). Data are expressed as mean±S.E.M. and analyzed with two-way ANOVA with three to four mice per experimental group.

We next asked if the profound late inhibition of neurogenesis in the absence of p53 after irradiation could be due to increased ablation of neural stem cells. We first characterized change in type-1 cell population (nestin+/GFAP+ or SOX2+/GFAP+ cells) in *p53*+/+ mice at 9 weeks after irradiation. Animals were given BrdU daily for 7 days at 4 weeks after irradiation for cell fate tracing. About half of the nestin+/GFAP+ cells (361±38, 17 Gy *versus* 693±30, 0 Gy; *P*<0.01, *t*-test) and SOX2+/GFAP+ cells (123±10, 17 Gy *versus* 289±530 Gy, 0 Gy; *P*<0.01) disappeared at 9 weeks after 17 Gy. Newborn type-1 cells (BrdU+/nestin+/GFAP+ cells; Figures 3a–d) showed a dose-dependent ablation after irradiation (0 Gy, 70.0±10.1; 5 Gy, 23.4±11.6; 17 Gy, none observed, *P*<0.005; one-way ANOVA).

We next performed a population analysis of type-1 cells in *p53*+/+ and *p53*−/− mice at 9 weeks after 0 and 5 Gy using the same BrdU-labeling paradigm. A 5-Gy dose was used as it resulted in the loss of approximately half of the number of newborn neurons at 9 weeks, and was considered optimal to discern the effect of p53 or the lack of it. In non-irradiated mice, *p53 *genotype had no effect on the total number of type-1 cells, BrdU+ (newborn) type-1 cells and Ki67+ (proliferating) type-1 cells (Figures 3e–h). Increased ablation of total, newborn and proliferating type-1 cells was observed in *p53*−/− mice compared with *p53*+/+ mice after irradiation (total type-1 cells: irradiation, *P*<0.001; *p53* genotype, *P*<0.05; BrdU+ type-1 cells: irradiation, *P*<0.05; *p53* genotype, *P*<0.001; Ki67+ type-1 cells: irradiation, *P*<0.001; *p53 *genotype, *P*<0.005, two-way ANOVA, Figures 3i–k). See Supplementary Table 1 for results of pairwise comparisons.

We did not observe any BrdU+/nestin+ cells that were non-type-1 cells in any control or irradiated *p53*+/+ and *p53*−/− mice. BrdU+ cells immunoreactive for Mash1, another marker of INPs, were also not observed. These results were consistent with culling and/or differentiation of INPs over the 5 weeks after they incorporated BrdU.^20^ Taken together, these results are consistent with increased neural stem cell exhaustion in *p53*−/− mice after irradiation.

### P53 regulates neural stem cell and progenitor cell fate after irradiation

To determine if dysregulated neural stem cell and NPC fate underlies the increased inhibition of neurogenesis associated with p53 deficiency after radiation, a single dose of BrdU (150 mg/kg) was given at 4 weeks after 0 or 5 Gy, and the number of type-1, -2 and -3 cells in *p53*+/+ and *p53*−/− mice was determined at 2 h, 2 days, 1 week and 5 weeks after BrdU administration. Using these schemas, BrdU+ cells at 2 h represented proliferating cells, those at 2 days a blend of proliferating and newly divided cells and those at 1 and 5 weeks were principally cells born during the 1- and 5-week interval, respectively, after BrdU administration.

**Figure 4:**
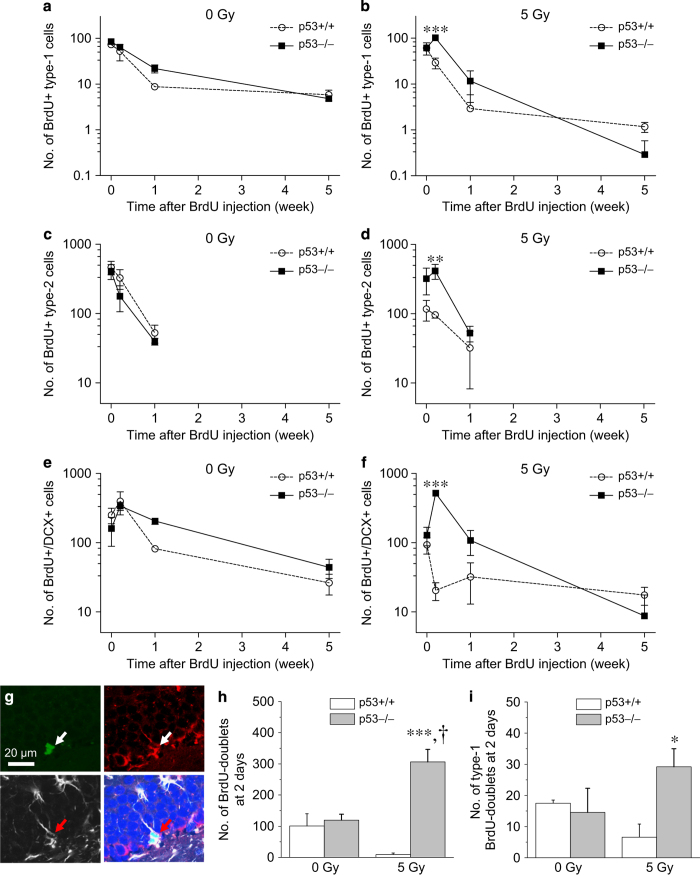
Deficiency in p53 alters neural stem cell and progenitor cell fate after irradiation. In non-irradiated mice, *p53* genotype does not alter the decline of BrdU+ type-1 cells over time after BrdU (**a**). After 5 Gy, the decrease in the number of BrdU+ type-1 cells over time is p53 dependent (**b**). The decline of BrdU+ type-2 cells over time is independent of *p53* genotype in non-irradiated mice (**c**) and is *p53* genotype dependent after 5 Gy (**d**). The number of BrdU+/DCX+cells over time after BrdU is independent of *p53* genotype in non-irradiated mice (**e**) but *p53* genotype dependent after 5 Gy (**f**). A type-1 BrdU-doublet is observed in SGZ of a *p53*−/− mouse after irradiation (**g**, arrow; BrdU, green; nestin, red; GFAP, white). The number of BrdU doublets and type-1 BrdU doublets in SGZ at 2 days after BrdU is *p53* genotype dependent following 5 Gy (**h** and **i**). BrdU was given at 4 weeks after 0 or 5 Gy, and cell populations determined at 2 h, 2 days, 1 and 4 weeks after BrdU. Data are represented as mean±S.E.M. and analyzed with two-way ANOVA and *post hoc* Bonferroni test, *P<0.05, **P<0.01, ***P<0.001, *p53*−/− *versus p53*+⧸+; ^†^*P*<0.01, 5 Gy *versus* 0 Gy in *p53*−/− mice. There was a minimum of three to four mice per genotype per time point.

In non-irradiated mice, the number of BrdU+ type-1 (BrdU+/nestin+/GFAP+) cells declined over the 5 weeks after BrdU but *p53* genotype had no effect on the cell numbers (time after BrdU, *P*<0.0001; *p53* genotype*, P*-value not significant; two-way ANOVA; Figure 4a). In contrast, the number of BrdU+ type-1 cells after 5 Gy was *p53 *genotype dependent (time after BrdU, *P*<0.0001; *p53* genotype, *P*<0.05; interaction, *P*<0.005; Figure 4b). Irradiation resulted in a spike of BrdU+ type-1 cells in *p53*−/− mice at 2 days after BrdU compared with *p53*+/+ mice (*P*<0.001, Bonferroni *post hoc* analysis (Figure 4b), see Supplementary Table 1 for results of pairwise comparisons). Hence, neural stem cell fate was not altered by *p53* genotype in the absence of irradiation, but there was enhanced activation in the absence of p53 after irradiation.

The number of BrdU+ type-2 cells (BrdU+/nestin+/GFAP− cells) decreased rapidly by 2 days and 1 week after BrdU in both non-irradiated *p53*+/+ and *p53*−/− mice (time after BrdU, *P*<0.0001; *p53* genotype*, P*-value not significant; Figure 4c). Irradiation resulted in an increase in BrdU+ type-2 cells at 2 days in *p53*−/− compared with *p53*+/+ mice (*P*<0.01; Figure 4d), and *p53 *genotype had a significant effect in the number of BrdU+ type-2 cells observed after irradiation (time after BrdU, *P*<0.005; *p53* genotype*, P*<0.05; interaction, *P*<0.05; Figure 4d). No BrdU+ type-2 cells were identified at 5 weeks after BrdU in control or irradiated mice irrespective of *p53* genotype.

In non-irradiated mice, BrdU+/DCX+ cells declined over 5 weeks after BrdU and *p53* genotype had no effect (time after BrdU, *P*<0.0005; *p53*, *P*-value not significant; Figure 4e). After irradiation, BrdU+/DCX+ cells also showed an increase at 2 days in *p53*−/− mice compared with *p53*+/+ mice after 5 Gy (*P*<0.001; Figure 4f). This was followed by decline over the next 5 weeks with *p53 *genotype demonstrating a significant effect (time after BrdU, *P*<0.0001; *p53* genotype, *P*<0.0001; interaction, *P*<0.0001; Figure 4f).

For further evidence of enhanced NPC renewal in *p53*−/− mice after irradiation, we determined the number of BrdU doublets and type-1 (nestin+/GFAP+) BrdU doublets (Figure 4g) at 2 days after BrdU. In the absence of irradiation, there was no difference in the number of BrdU doublets in *p53*+/+ mice compared with *p53*−/− mice. After 5 Gy, the number of BrdU doublets decreased in *p53*+/+ mice but increased in *p53*−/− mice (Figure 4h). Similar observations were noted for type-1 BrdU doublets (Figure 4i). Thus, p53 does not alter neural stem cell fate in non-irradiated hippocampus, but absence of p53 results in enhanced activation and renewal after irradiation.

### P53 deficiency does not alter neuroinflammation or neurovascular niche dysfunction after irradiation

**Figure 5:**
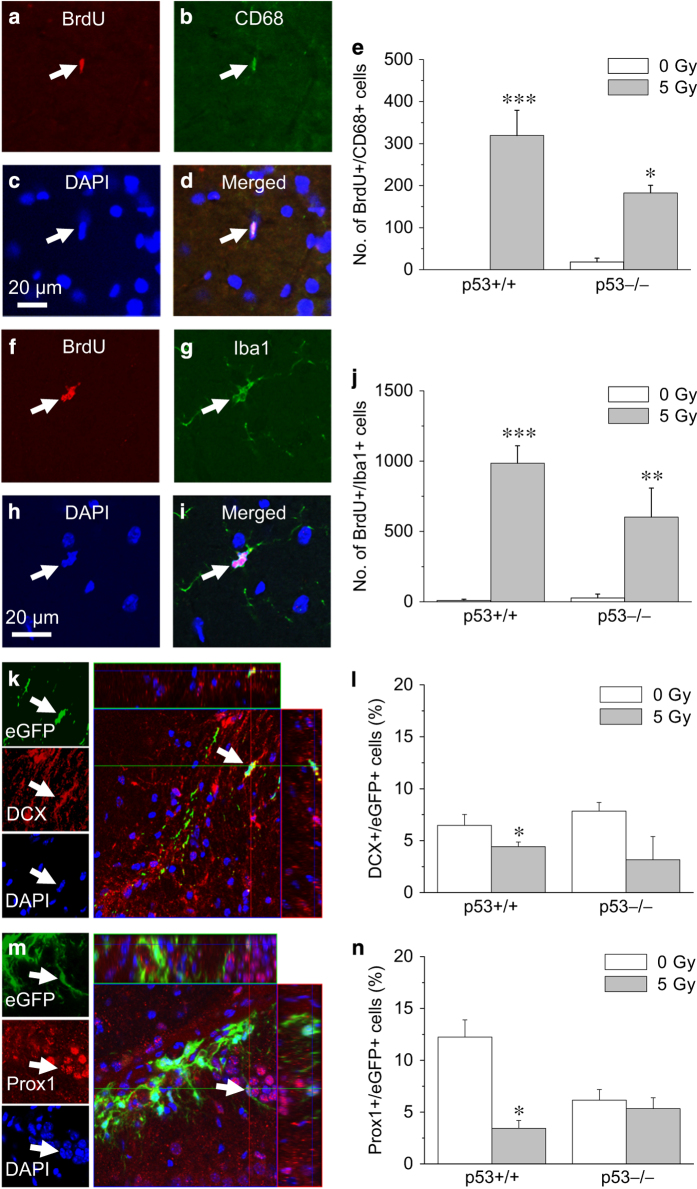
Deficiency in p53 does not alter microglial activation or inhibition of neuronal differentiation after irradiation. An activated microglia demonstrates nuclear BrdU incorporation and CD68+ (**a–d**, arrow) or Iba1+ (**f–i**, arrow). The increase in the number of BrdU+/CD68+ (**e**) and BrdU+/Iba1+ (**j**) cells in the dentate gyrus at 9 weeks after cranial irradiation is independent of *p53* genotype. An eGFP+ neural progenitor cell transplanted in mouse hippocampus demonstrates immunoreactivity for DCX (**k**, arrow) and another one for Prox1 (**m**, arrow). The percentage of eGFP+ cells that expresses DCX or Prox1 is reduced in mice given cranial irradiation before transplantation, independent of *p53* genotype of the recipient mice (**l**, DCX+/eGFP+ cells; **n**, Prox1+/eGFP+ cells). Data are expressed as mean±S.E.M. and analyzed with two-way ANOVA and *post hoc* Bonferroni test, *P<0.05, **P<0.01, ***P<0.001, 5 Gy *versus* 0 Gy; a minimum of three to five mice per experimental group (**e **and **j**) and four to seven mice per experimental group (**l **and **n**).

The fate of neural stem cells and NPCs is regulated by neurovascular interactions.^23^ Damage to the neurogenic niche such as neuroinflammation is thought to contribute to the deficit in neurogenesis after irradiation.^8,24–28^ We thus asked whether the increased inhibition of neurogenesis after irradiation in p53-deficient mice could also be related to increased microglial activation after irradiation. Newborn microglia (BrdU+/CD68+ and BrdU+/Iba1+ cells; Figures 5a–i) have been extensively used as surrogates for activated microglia.^26,29,30^ Nine weeks after 5 Gy (BrdU given daily for 7 days at 4 weeks after irradiation), there was an increase in BrdU+/CD68+ and BrdU+/Iba1+ cells in dentate gyrus, independent of *p53* genotype (BrdU+/CD68+ cells: radiation, *P*<0.0001; *p53* genotype, *P*-value not significant; BrdU+/Iba1+ cells: radiation, *P*<0.0001; *p53* genotype, *P*-value not significant; two-way ANOVA; Figures 5e and j).

To examine whether there was increased damage of the neurogenic niche after irradiation in the absence of the p53, and hence its ability to support neurogenesis, we asked if there could be increased inhibition of neuronal differentiation of NPCs transplanted into irradiated *p53*−/− mouse hippocampus compared with irradiated *p53*+/+ mouse hippocampus. *P53*+/+ and *p53*−/− mice were given 0 or 5 Gy. After 3 weeks, NPCs cultured from the hippocampus of enhanced green fluorescent protein (eGFP) mice were stereotactically transplanted into the hippocampus.^19^ At 5 weeks after transplantation, eGFP cells immunoreactive for DCX or Prox1 could be seen in the hippocampus (Figures 5k and m). Only the rare NeuN+/eGFP+ cells were found. The proportion of eGFP cells that expressed DCX or Prox1 was decreased in irradiated hippocampus compared with control, but *p53* genotype had no effect (DCX+ cells: irradiation, *P*<0.01; *p53* genotype, *P*-value not significant; Prox1+ cells: irradiation, *P*<0.005; *p53* genotype, *P*-value not significant; two-way ANOVA; Figures 5l and n). These results did not support the notion that the irradiated microenvironment in *p53*−/− hippocampus had further inhibitory effects on neuronal differentiation compared with wild-type mice. Taken together, the increase in disruption of neurogenesis in *p53*−/− mice after irradiation is unlikely to be due to increased microglial activation or increased injury in the irradiated *p53*−/− neurogenic niche.

## Discussion

The adult mammalian brain contains neural stem cells that have the ability to proliferate and generate multipotential NPCs that differentiate into neurons.^3,31^ Although neural stem cells are able to proliferate, their capacity for self-renewal is finite. Fate mapping studies revealed that a type-1 cell upon exiting its quiescent state undergoes only a few rounds of asymmetric divisions to produce mature neurons and self-renew.^32^ Division coupled production of new neurons is thought to result in age-related depletion of the neural stem cell pool.^33,34^

We observed depletion of total, proliferating and newborn type-1 cells after irradiation. Their ablation after irradiation was further enhanced in the absence of p53. There was an increase in the number of BrdU+ type-1 cells and type-1 BrdU doublets at 2 days after BrdU given 4 weeks after 5 Gy, whereas the opposite effect was seen in *p53*+/+ mice. Hence, the absence of p53 resulted in enhanced neural stem cell activation after irradiation, whereas neural stem cell fate did not appear to be altered by p53 in non-irradiated mice.

During neurogenesis in adult dentate gyrus, only a few newborn cells become mature neurons. The majority of newborn die of apoptosis within a few days of birth before they transition into DCX+ neuroblasts.^20^ In non-irradiated mice, regardless of *p53* genotype, we also observed a sharp decline in the number of BrdU+ type-2 and BrdU+/DCX+ cells between 2 and 7 days after BrdU.

A homeostasis of neural stem cell activation and quiescence allows for the continuous generation of new neurons throughout life. Disruption of signaling pathways that lead to excessive activation of neural stem cells resulted in their subsequent depletion and failure of neurogenesis.^33,35,36^ Certain brain pathologies such as seizures and trauma associated with activation of stem cell division also demonstrated their accelerated loss.^37,38^ P53 is known to negatively regulate NPC proliferation *in vitro*. Based on the neurosphere assay, it was postulated p53 might negatively regulate self-renewal of neural stem cells.^15^ In a previous study, *p53*−/− mice were noted to have accelerated ‘neurogenesis’ in dentate gyrus within 2 weeks after irradiation based on the expression of cyclin-dependent kinase 1.^39^ Our *in vivo* data here showed that p53 deficiency did not alter neural stem cell fate in non-irradiated hippocampus. Enhanced neural stem cell activation associated with p53 deficiency was only observed after irradiation. Given the well-known effect of ionizing radiation in mitotic-linked death, we propose that in the absence of p53, increased cell cycle entry leads to enhanced division coupled death and consequential depletion of neural stem cell pool and profound inhibition of neurogenesis.

For the cell fate studies, our results were unlikely to be confounded by the potential dilution of the BrdU labeling over the 5-week interval since the two genotypes were compared at the same time point. It might be argued if *p53*−/− NPCs undergo more divisions than *p53*+/+ NPCs, there could be increased dilution of the BrdU label below the level of detection to yield lower counts of BrdU-retained cells in irradiated *p53*−/− mice. This is however not supported by the greater number of BrdU+/NeuN+ cells in non-irradiated *p53*−/− dentate gyrus compared with wild-type mice. Recent studies on hippocampal neurogenesis using similar BrdU paradigms reported negligible impact of label dilution up to 30 days after BrdU injections.^20,34^

How p53 regulates the differential DNA damage response in neural stem cells and NPCs remains unclear. We showed here that *p53*−/− NPCs *in vitro* demonstrated a slower clearance of *γ*H2AX foci compared with *p53*+/+ cells. For hematopoietic and mammary stem cells *in vitro*, DNA damage resulted in the activation of p21 and inhibition of p53, which lead to cell cycle entry and symmetric self-renewing divisions.^40^ The increase in BrdU-labeled type-1 cells at 2 days after BrdU in irradiated *p53*−/− mice compared with wild-type mice is consistent with stem cell activation and symmetric division. Considerable heterogeneity, however, exists in the DNA damage response of tissue-specific stem cells.^41^

Endothelium deficient of *p53* gene has been noted to have increased radiosensitivity.^13^ P53-regulated responses mediated by endothelium may modulate late normal tissue responses after radiation treatment. Mice with endothelial cell-specific deletion of *p53* demonstrated increased 2-month lethality from gastrointestinal syndrome after subtotal body irradiation.^11^ Endothelial cell-specific deletion of *p53* was also shown to result in increased myocardial injury after whole-heart irradiation.^12^ Endothelial cells represent a key component of the neurogenic niche.^23^ There is an intimate association of hippocampal neurogenesis with angiogenesis.^42^ Inhibition of neurogenesis is associated with increased microglial activity, and reducing neuroinflammation has been shown to partially restore deficit in neurogenesis after irradiation.^8,9,25–28^ Disruption of the neurogenic niche is thought to contribute to failure of NPCs to differentiate into neuroblasts after irradiation. Here we observed no evidence of increased microglial activation in irradiated *p53*−/− mice compared with *p53*+/+ mice. Similarly, results of the transplantation experiment failed to demonstrate increased failure of neuronal differentiation of NPCs in irradiated *p53*−/− mice. These results were consistent with the BrdU cell fate study, which showed an increase rather than decrease in newborn/proliferating DCX+ cells in *p53*−/− dentate gyrus 4 weeks after irradiation compared with irradiated wild-type mice. Hence, the increased disruption of neurogenesis after irradiation in the absence of p53 is unlikely to be due to increased damage of the *p53*−/− neurogenic niche after irradiation.

Abrogating apoptosis has been shown to augment adult neurogenesis.^6^ Pharmacologic approaches to suppress apoptosis have thus been proposed as potential therapeutic strategies to mitigate radiation-induced inhibition of neurogenesis.^43,44^ Radiation-induced apoptosis of NPCs in the dentate gyrus is abrogated in the absence of p53.^19,21,22^ Here we observed profound inhibition of neurogenesis in irradiated *p53*−/− hippocampus that failed to mount an apoptotic response in NPCs. These results provide compelling evidence that p53 regulates neuronal development independent of apoptosis of neuroblasts after irradiation.

In summary, deficiency in p53 resulted in profound inhibition of adult neurogenesis after irradiation independent of apoptosis. There was no evidence of increased neuroinflammation and damage of the neurogenic niche in *p53*−/− hippocampus after irradiation. Rather, p53 deficiency resulted in increased activation of neural stem cells and NPCs after irradiation, leading to subsequent exhaustion of the neural stem cell pool. We propose that p53 serves to mitigate disruption of neuronal development after irradiation and may thus have a role in regulating late effects in brain after irradiation.

## Materials and Methods

### Animals

Ten-week-old male C57 mice +/+, +/− or −/− for *p53* (Jackson Laboratory, Bar Harbor, ME, USA) were irradiated as described previously.^19^ *P53*^*S*^ mice were generous gifts from Dr. Manual Serrano, and have one extra copy of the normal *p53* gene.^17^ NPCs for transplantation were cultured from the brain of *Tg/CAG-EGFP/B5Nagy* mice (Jackson Laboratory) that express eGFP.^19^ They were wild type for *p53*. Mouse colonies were maintained by littermate inbreeding, housed under a 12–12 h light–dark cycle at 21 °C and fed a standard rodent diet with food and water *ad libitum*. Genotyping was performed by PCR as described previously.^19^ Only male mice were used to avoid the potential confounding influence of sex and estrous cycles on neuronal development.^45^ All animal protocols were approved by the institutional animal care committee in accordance with the Canadian Council on Animal Care guidelines.

### Irradiation

Animals were anesthetized using an intraperitoneal injection of ketamine (75 mg/kg) and xylazine (6 mg/kg), immobilized in a customized jig, and the entire hippocampus was irradiated using an anterior–posterior and posterior–anterior pair of 160 kV X-ray beam (CP160, Faxitron X-ray) defined by an 8-mm diameter lead cut-out.^19^

### BrdU incorporation

Various BrdU incorporation schedules were used for cell fate mapping as described in the Results section. BrdU was administered by intraperitoneal injection.

### Primary culture of NPCs

Neurospheres were cultured from 8-week-old *p53*+/+, *p53*−/− and eGFP mouse hippocampus.^19^ After 10 days in culture, mechanically dissociated neurosphere cells were plated onto culture slips precoated with poly-l-ornithine (Sigma-Aldrich, St Louis, MO, USA) and fed with DMEM/F12 medium containing penicillin/streptomycin, B27 supplement, basic fibroblast growth factor and epidermal growth factor. The non-differentiation medium was changed every other day until cells grew to confluence on day 8. NPCs cultured from *p53*+/+, *p53*−/− and eGFP mice demonstrated multipotential properties as reported previously.^19^

### Transplantation of eGFP-NPCs

eGFP-NPCs after 8 days in culture were dissociated into single-cell suspensions in DMEM/F12 medium, and stored in ice before transplantation. Transplantation was carried out within 3 h following cell harvesting. eGFP-NPCs were transplanted into the hippocampus of *p53*+/+ and *p53*−/− mice, which had received 0 or 5 Gy of cranial irradiation 3 weeks previously. The cranium was fixed in a stereotactic frame (Kopf Small Animal Stereotaxtic 900) during transplantation with the animals under anesthesia using a cocktail of ketamine and xylazine.^19^ Two craniotomies were performed to allow cell transplantation into the right dentate gyrus in two locations: first location, 1.8 mm laterally to the right, 1.1 mm caudally and 3.3 mm ventrally; second location, 2.6 mm laterally to the right, 1.6 mm caudally and 3.6 mm ventrally, all with reference to the bregma.

A suspension of 2.5 *μ*l of eGFP cells (50 000 cells per *μ*l) in DMEM/F12 medium was introduced at 1 *μ*l/min into each transplantation site, and for an additional 2 min to allow pressure equalization. The scalp was closed with synthetic suture monofilament after transplantation. Subcutaneous buprenorphin (0.05–0.1 mg/kg) was given as applicable. Antibiotics were not used.^19^

### Histopathology and immunohistochemistry

Under anesthesia with ketamine and xylazine, mice were perfused with 0.9% saline followed by 4% paraformaldehyde in PBS. Mouse brains were retrieved, postfixed for 2 days and cryoprotected in a 30% sucrose solution. Coronal sections between 1.3 and −3.5 mm caudal to the bregma were cut at 40-*μ*m thickness, collected in tissue cryoprotectant solution in 96-well plates and stored at *−*20 °C before immunohistochemistry.

As morphological characterization remains the gold standard for identification of apoptotic cells,^20^ cells that showed nuclear condensation and fragmentation upon 4′,6-diamidino-2-phenylindole (DAPI) staining were considered apoptotic cells.^19^ Apoptotic cells were further identified and quantified using TUNEL and caspase-3 (1 : 1000; Cell Signaling Technology, Beverly, MA, USA) immunohistochemistry.^19^

NPCs, immature and mature neurons and microglia were identified by different phenotypic markers using antibodies listed in Supplementary Table 2. Secondary antibodies were conjugated to Cy2, Cy3 (1 : 200; Jackson ImmunoResearch, West Grove, PA, USA) or Alexa Fluor 647 (1 : 200; Invitrogen, Waltham, MA, USA). Colocalization of BrdU (1 : 200; Abcam, Toronto, ON, Canada), Ki67 (1 : 1000; Novocastra, Newcastle upon Tyne, UK ) and phenotypic markers in selected sections were evaluated using a confocal laser scanning microscope (Zeiss LSM700, Carl Zeiss AG Corporate, Oberkochen, Germany). A BrdU-doublet was defined as two abutting DAPI-stained nuclei that demonstrated nuclear BrdU immunoreactivity.

### Stereological analysis

Apoptotic cells and cells labeled using different phenotypic markers were counted within the dentate gyrus including a 50-*μ*m hilar margin of the SGZ.^19^ Cell counting was performed using a Zeiss Imager M1 microscope (Carl Zeiss AG Corporate) with the Stereo Investigator software (MBF Bioscience, Williston, VT, USA). The observers were blinded to the experimental groups. Apoptotic cells were counted using a counting frame and a sampling grid of 75×75 *μ*m^2^, NPCs using counting frame of 20×20 *μ*m^2^ and sampling grid of 180×180 *μ*m^2^, and microglia, counting frame and sampling grid of 75×75 *μ*m^2^, all at a magnification of ×630. Every seventh section was used as the periodicity of sections sampled.

For the transplantation study, 10 coronal sections containing the hippocampus at 5-section intervals from each mouse were used for exhaustive cell counting of eGFP cells with a 100×100 *μ*m^2^ sampling grid. The coefficient of error for all the stereology data was between 0.03 and 0.06.

### Assessment of DNA damage repair foci

NPCs from *p53*+/+ and *p53*−/− mice were cultured in non-differentiation medium for 8 days before they were given a single dose of 0 or 5 Gy. At various time intervals up to 24 h after irradiation, cells were fixed with 4% paraformaldehyde for 10 min at room temperature. After treatment with 0.5% nonylphenoxypolyethoxylethanol in PBS, sections were incubated with mouse anti-phospho-histone H2AX IgG1 antibody (1 : 200; Millipore, Billerica, MA, USA) at 4 °C overnight followed by donkey anti-mouse Cy3 for 45 min at room temperature, and counterstained with DAPI. A minimum of 50 nuclei from a minimum of five independent experiments per treatment group was used to determine the number of *γ*H2AX foci per nucleus. As the occasional non-irradiated NPC nuclei contained up to six foci, the nuclei with ≥5 foci were considered foci+.

### Statistical analysis

All cell population analysis represented data from three to five mice per genotype per dose per time point, except for the cell fate experiments where there were three to four mice per genotype per dose group. There were four to seven mice per experimental group in the transplantation experiment. All data were expressed as mean±S.E. Comparison of cell numbers after irradiation to controls was performed using *t*-test. Dose–response analysis for cell numbers was performed by one-way ANOVA. The effect of variables, namely irradiation and *p53* genotype, or *p53* genotype and time after BrdU on cell numbers, was determined using two-way ANOVA. Pairwise comparisons were based on *post hoc* Bonferroni correction for multiple comparisons. Differences were considered significant for *P*<0.05. Statistical analyses were performed with the GraphPad Prism 5 (GraphPad Software, La Jolla, CA, USA).

## Abbreviations

ANOVA: analysis of variance
BrdU: bromodeoxyuridine
DAPI, 4′: 6-diamidino-2-phenylindole
DCX: doublecortin
eGFP: enhanced green fluorescent protein
GFAP: glial fibrillary acidic protein
INP: intermediate neural progenitors
NeuN: neuronal nuclei
NPCs: neural progenitor cells
+: positive
SGZ: subgranular zone
SOX2: sex-determining region Y-box 2
TUNEL: terminal deoxynucleotidyl transferase-mediated dUTP nick-end labeling.
