# Supplementary material for: P53 regulates disruption of neuronal development in the adult hippocampus after irradiation
Source: Cell Death Discov. 2016 Oct 3;2:16072–. doi: 10.1038/cddiscovery.2016.72 (PMC5045962; doi:10.1038/cddiscovery.2016.72)
Supplement: Supplementary Table 1 [file cddiscovery201672-s1.doc]

| **Supplementary Table 1.**  Results of pair wise comparison on cell populations based on two-way ANOVA and post-hoc Bonferroni analysis. Only results with *P* < 0.05 are listed. | | | | |  |
| --- | --- | --- | --- | --- | --- |
|  | | |  | | |
|  |  |  | |  | |
| **Figure 1n** |  |  | |  | |
| No. of BrdU+/NeuN+ cells after single doses |  |  | |  | |
| p53+/+ 0 Gy vs. p53+/– 0 Gy | P < 0.05 |  | |  | |
| p53+/+ 0 Gy vs. p53–/– 0 Gy | P < 0.001 |  | |  | |
| p53+/– 0 Gy vs. p53+/– 5 Gy | P < 0.001 |  | |  | |
| p53+/– 0 Gy vs. p53+/– 10 Gy | P < 0.001 |  | |  | |
| p53+/– 0 Gy vs. p53+/– 17 Gy | P < 0.001 |  | |  | |
| p53+/– 0 Gy vs. p53–/– 0 Gy | P < 0.001 |  | |  | |
| p53–/– 0 Gy vs. p53–/– 5 Gy | P < 0.001 |  | |  | |
| p53–/– 0 Gy vs. p53–/– 10 Gy | P < 0.001 |  | |  | |
| p53–/– 0 Gy vs. p53–/– 17 Gy | P < 0.001 |  | |  | |
|  |  |  | |  | |
| **Figure 1o** |  |  | |  | |
| No. of BrdU+/NeuN+ cells after 20 Gy in 5 fractions | |  | |  | |
| p53+/+ 0 Gy vs. p53+/+ 20 Gy | P < 0.001 |  | |  | |
| p53+/+ 0 Gy vs. p53–/– 0 Gy | P < 0.001 |  | |  | |
| p53+/– 0 Gy vs. p53+/– 20 Gy | P < 0.001 |  | |  | |
| p53+/– 0 Gy vs. p53–/– 0 Gy | P < 0.01 |  | |  | |
| p53–/– 0 Gy vs. p53–/– 20 Gy | P < 0.001 |  | |  | |
|  |  |  | |  | |
| **Figure 2m** |  |  | |  | |
| No. of TUNEL+/DCX+ cells |  |  | |  | |
| p53+/+ 0 Gy vs. p53+/+ 5 Gy | P < 0.001 |  | |  | |
| p53+/+ 0 Gy vs. p53+/+ 17 Gy | P < 0.001 |  | |  | |
|  |  |  | |  | |
| **Figure 3i** |  |  | |  | |
| No. of total type-1 cells |  |  | |  | |
| p53–/– 0 Gy vs. p53–/– 5 Gy | P < 0.05 |  | |  | |
|  |  |  | |  | |
| **Figure 3j** |  |  | |  | |
| No. of BrdU+ type-1 cells |  |  | |  | |
| p53+/+ 0 Gy vs. p53+/+ 5 Gy | P < 0.001 |  | |  | |
| p53–/– 0 Gy vs. p53–/– 5 Gy | P < 0.01 |  | |  | |
|  |  |  | |  | |
| **Figure 3k** |  |  | |  | |
| No. of Ki67+ type-1 cells |  |  | |  | |
| p53+/+ 0 Gy vs. p53+/+ 5 Gy | P < 0.01 |  | |  | |
| p53–/– 0 Gy vs. p53–/– 5 Gy | P < 0.01 |  | |  | |
|  |  |  | |  | |
| **Figure 4a** |  |  | |  | |
| No. of BrdU+ type-1 cells after 0 Gy |  |  | |  | |
| p53+/+ 2 hours vs. p53+/+ 1 week | P < 0.001 |  | |  | |
| p53+/+ 2 hours vs. p53+/+ 4 weeks | P < 0.001 |  | |  | |
| p53+/+ 2 days vs. p53+/+ 1 week | P < 0.01 |  | |  | |
| p53+/+ 2 days vs. p53+/+ 4 weeks | P < 0.01 |  | |  | |
| p53–/– 2 hours vs. p53–/– 1 week | P < 0.001 |  | |  | |
| p53–/– 2 hours vs. p53–/– 4 weeks | P < 0.001 |  | |  | |
| p53–/– 2 days vs. p53–/– 1 week | P < 0.01 |  | |  | |
| p53–/– 2 days vs. p53–/– 4 weeks | P < 0.001 |  | |  | |
|  |  |  | |  | |
| **Figure 4b** |  |  | |  | |
| No. of BrdU+ type-1 cells after 5 Gy |  |  | |  | |
| p53+/+ 2 hours vs. p53+/+ 1 week | P < 0.01 |  | |  | |
| p53+/+ 2 hours vs. p53+/+ 4 weeks | P < 0.01 |  | |  | |
| p53+/+ 2 days vs. p53–/– 2 days | P < 0.01 |  | |  | |
| p53–/– 2 hours vs. p53–/– 2 days | P < 0.05 |  | |  | |
| p53–/– 2 hours vs. p53–/– 1 week | P < 0.01 |  | |  | |
| p53–/– 2 hours vs. p53–/– 4 weeks | P < 0.01 |  | |  | |
| p53–/– 2 days vs. p53–/– 1 week | P < 0.001 |  | |  | |
| p53–/– 2 days vs. p53–/– 4 weeks | P < 0.001 |  | |  | |
|  |  |  | |  | |
| **Figure 4c** |  |  | |  | |
| No. of BrdU+ type-2 cells after 0 Gy |  |  | |  | |
| p53+/+ 2 hours vs. p53+/+ 1 week | P < 0.01 |  | |  | |
| p53+/+ 2 hours vs. p53+/+ 4 weeks | P < 0.001 |  | |  | |
| p53+/+ 2 days vs. p53+/+ 1 week | P < 0.05 |  | |  | |
| p53+/+ 2 days vs. p53+/+ 4 weeks | P < 0.05 |  | |  | |
| p53–/– 2 hours vs. p53–/– 1 week | P < 0.01 |  | |  | |
| p53–/– 2 hours vs. p53–/– 4 weeks | P < 0.01 |  | |  | |
|  |  |  | |  | |
| **Figure 4d** |  |  | |  | |
| No. of BrdU+ type-2 cells after 5 Gy |  |  | |  | |
| p53+/+ 2 days vs. p53–/– 2 days | P < 0.01 |  | |  | |
| p53–/– 2 hours vs. p53–/– 1 week | P < 0.05 |  | |  | |
| p53–/– 2 hours vs. p53–/– 4 weeks | P < 0.01 |  | |  | |
| p53–/– 2 days vs. p53–/– 1 week | P < 0.01 |  | |  | |
| p53–/– 2 days vs. p53–/– 4 weeks | P < 0.001 |  | |  | |
|  |  |  | |  | |
| **Figure 4e** |  |  | |  | |
| No. of BrdU+/DCX+ cells after 0 Gy |  |  | |  | |
| p53+/+ 2 days vs. p53+/+ 1 week | P < 0.01 |  | |  | |
| p53+/+ 2 days vs. p53+/+ 4 weeks | P < 0.01 |  | |  | |
| p53–/– 2 days vs. p53–/– 4 weeks | P < 0.01 |  | |  | |
|  |  |  | |  | |
| **Figure 4f** |  |  | |  | |
| No. of BrdU+/DCX+ cells after 5 Gy |  |  | |  | |
| p53+/+ 2 days vs. p53–/– 2 days | P < 0.001 |  | |  | |
| p53–/– 2 hours vs. p53–/– 2 days | P < 0.001 |  | |  | |
| p53–/– 2 hours vs. p53–/– 4 weeks | P < 0.05 |  | |  | |
| p53–/– 2 days vs. p53–/– 1 week | P < 0.001 |  | |  | |
| p53–/– 2 days vs. p53–/– 4 weeks | P < 0.001 |  | |  | |
| p53–/– 1 week vs. p53–/– 4 weeks | P < 0.05 |  | |  | |
