# Supplementary material for: P53 regulates disruption of neuronal development in the adult hippocampus after irradiation
Source: Cell Death Discov. 2016 Oct 3;2:16072–. doi: 10.1038/cddiscovery.2016.72 (PMC5045962; doi:10.1038/cddiscovery.2016.72)
Supplement: Supplementary Table 2 [file cddiscovery201672-s2.doc]

**Supplementary Table 2**

List of primary antibodies used

| Antibodies | Dilution | Company |
| --- | --- | --- |
| BrdU | 1:200 | Abcam, Cambridge, MA |
| Caspase-3 | 1:1000 | Cell Signaling Technology, Danvers, MA, USA |
| Calbindin | 1:1000 | Millipore, Billerica, MA, USA |
| Calretinin | 1:200 | Abcam |
| CD68 | 1:200 | Serotec, Kidlington, UK |
| DCX | 1:2000 | Abcam |
| GFAP | 1:200 | DakoCytomation, Copenhagen, Denmark |
| γH2AX | 1:100 | Millipore |
| Iba1 | 1:1000 | Abcam |
| Ki67 | 1:1000 | Novocastra, Newcastle, UK |
| Mash1 | 1:50 | Abcam |
| Nestin | 1:200 | Millipore |
| NeuN | 1:500 | Millipore |
| p21 | 1:50 | Abcam |
| p53 | 1:500 | Novocastra |
| Prox1 | 1:200 | Abcam |
| SOX2 | 1:150 | Abcam |
