# Supplementary material for: P53 regulates disruption of neuronal development in the adult hippocampus after irradiation
Source: Cell Death Discov. 2016 Oct 3;2:16072–. doi: 10.1038/cddiscovery.2016.72 (PMC5045962; doi:10.1038/cddiscovery.2016.72)
Supplement: Supplementary Figure 1 [file cddiscovery201672-s3.ppt]

## Slide 1
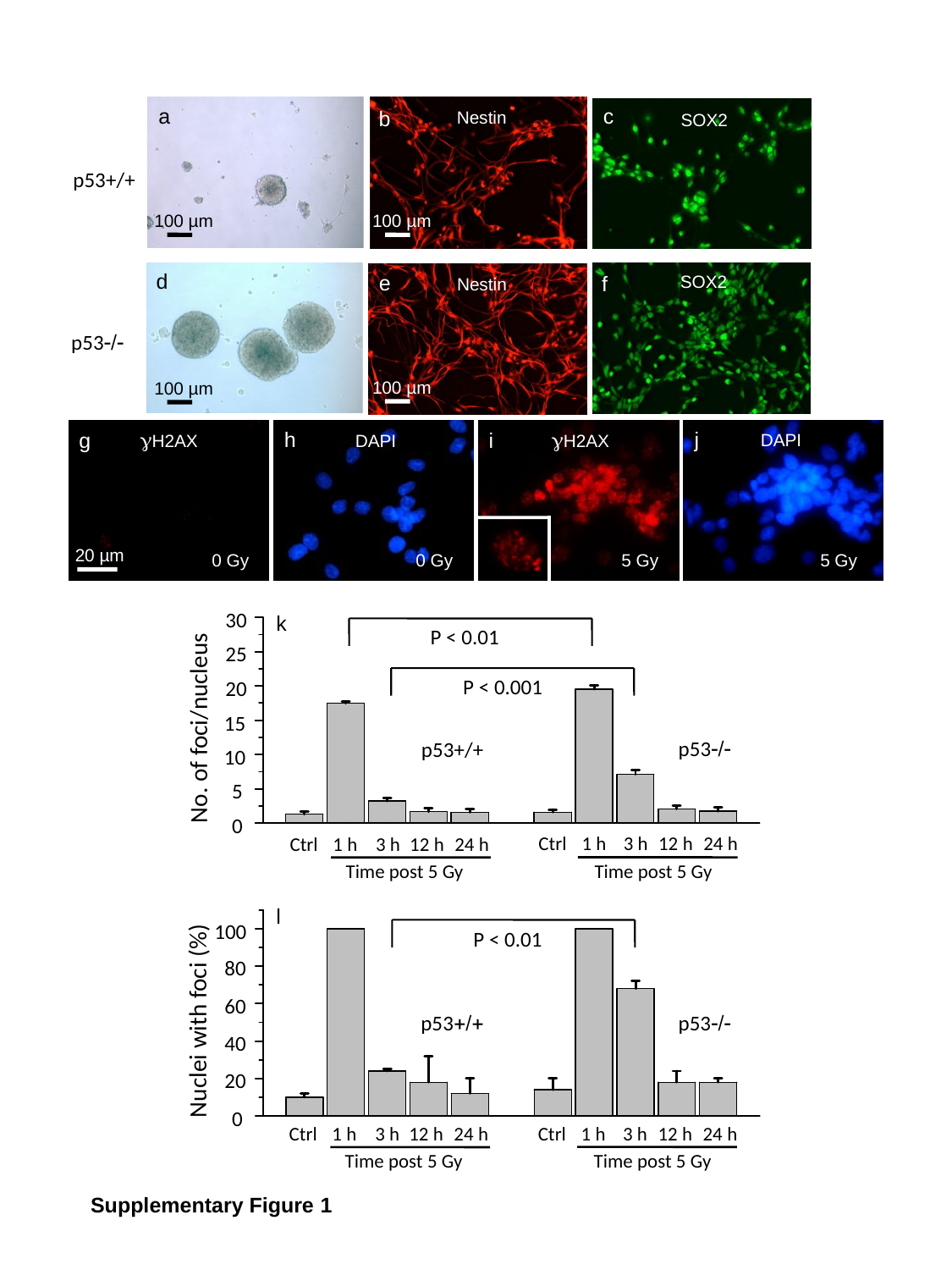

a
c
b
Nestin
SOX2
A
d
p53+/+
100 µm
100 µm
Nestin
e
f
SOX2
p53
100 µm
100 µm
γH2AX
h
γH2AX
j
g
i
DAPI
DAPI
20 µm
5 Gy
0 Gy
0 Gy
5 Gy
30
25
20
15
10
5
0
k
P < 0.01
P < 0.001
No. of foci/nucleus
p53
p53+/+
Ctrl
1 h
3 h
12 h
24 h
Ctrl
1 h
3 h
12 h
24 h
Time post 5 Gy
Time post 5 Gy
l
100
80
60
40
20
0
P < 0.01
Nuclei with foci (%)
p53
p53
Ctrl
1 h
3 h
12 h
24 h
Ctrl
1 h
3 h
12 h
24 h
Time post 5 Gy
Time post 5 Gy
Supplementary Figure 1
