# Supplementary figures and images for: P53 regulates disruption of neuronal development in the adult hippocampus after irradiation
Source: Cell Death Discov. 2016 Oct 3;2:16072–. doi: 10.1038/cddiscovery.2016.72 (PMC5045962; doi:10.1038/cddiscovery.2016.72)

## Slide 1
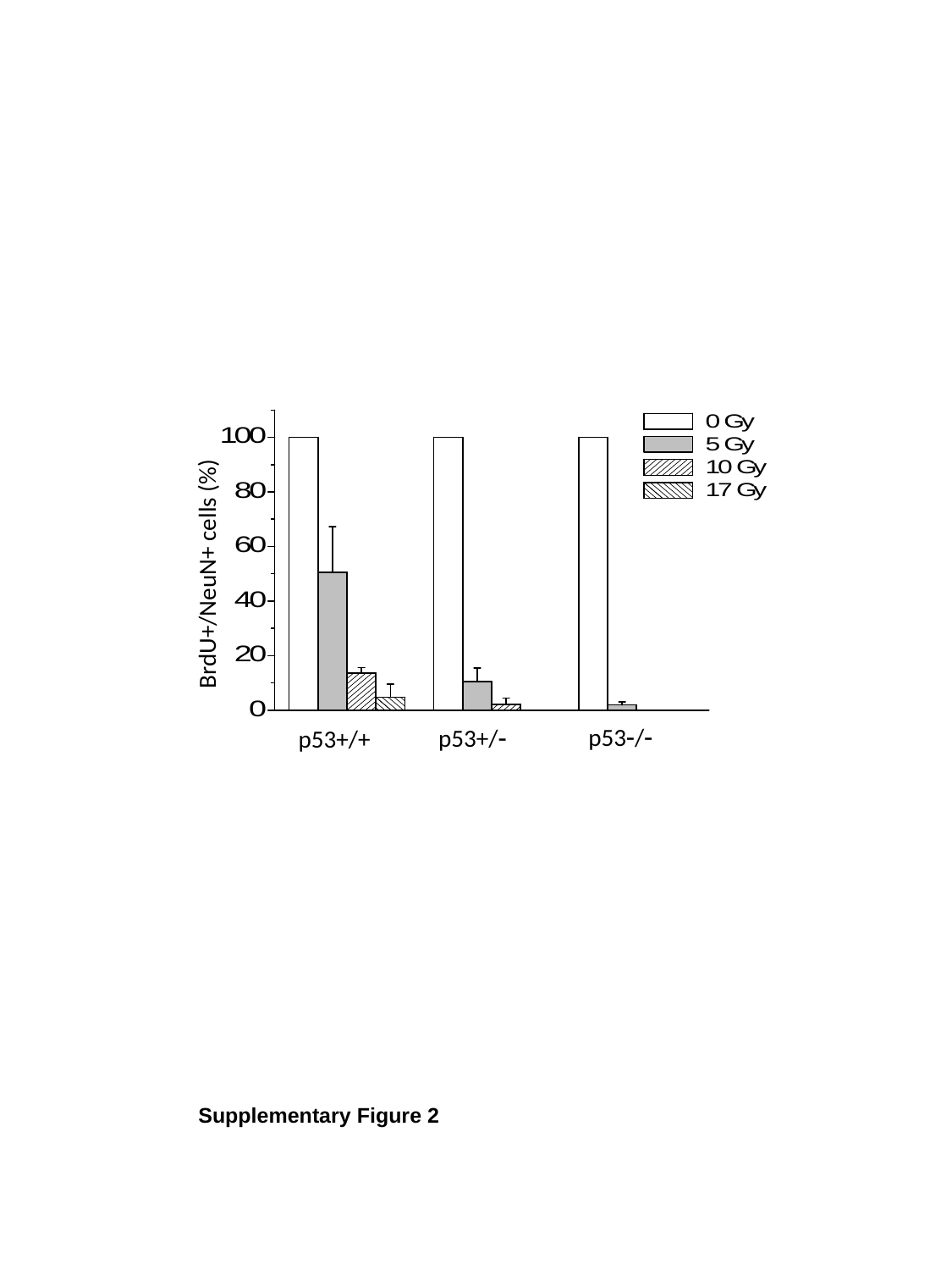

BrdU+/NeuN+ cells (%)
p53−/−
p53+/−
p53+/+
Supplementary Figure 2

Supplement: Supplementary Figure 2 [file cddiscovery201672-s4.ppt]

## Slide 1
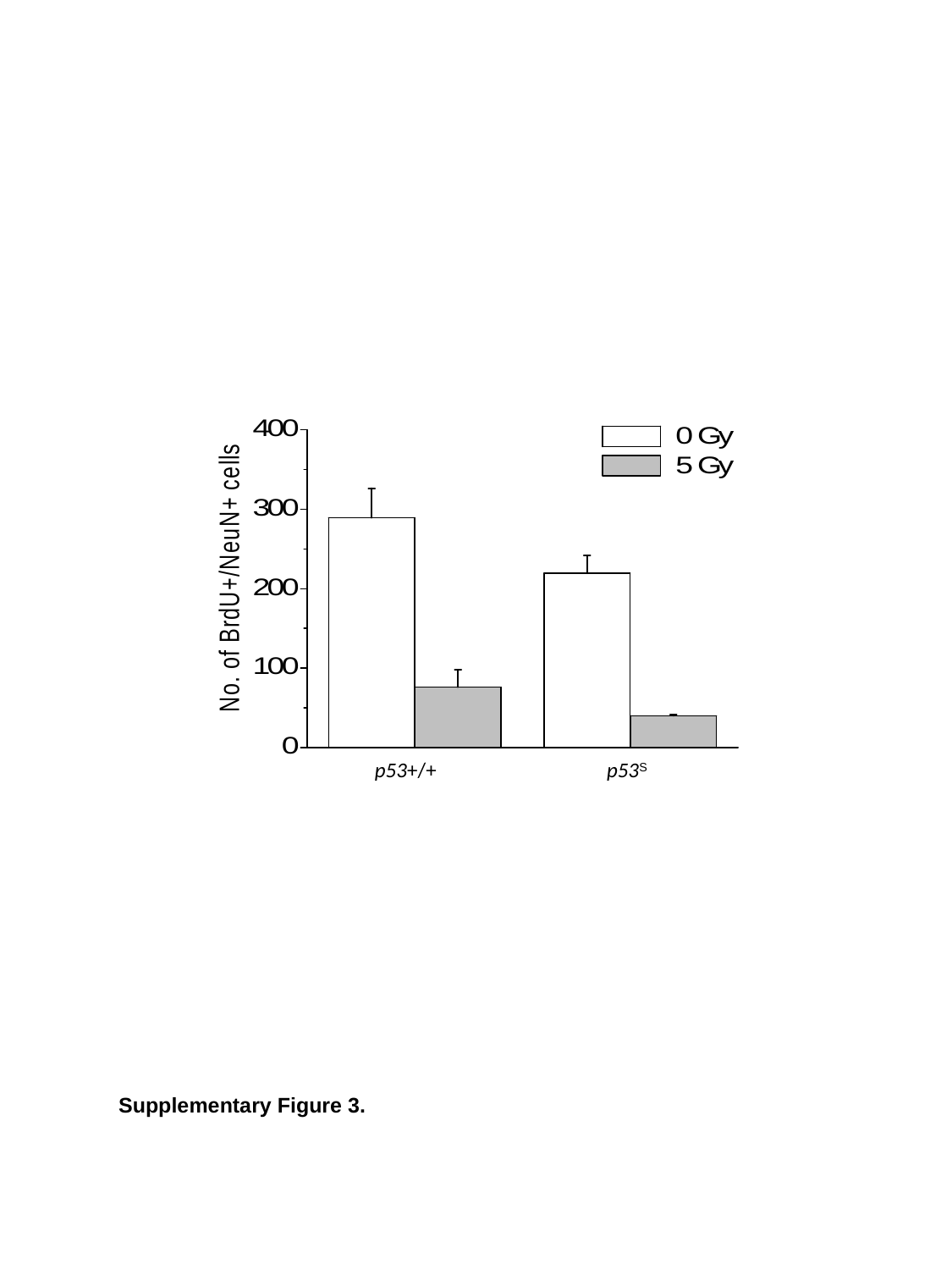

Pp53+/+ p53S
Supplementary Figure 3.

Supplement: Supplementary Figure 3 [file cddiscovery201672-s5.ppt]
